# How Per- and Poly-Fluoroalkyl Substances Affect Gamete Viability and Fertilization Capability: Insights from the Literature

**DOI:** 10.3390/jox14020038

**Published:** 2024-05-17

**Authors:** Cielle Lockington, Laura A. Favetta

**Affiliations:** Reproductive Health and Biotechnology Lab, Department of Biomedical Sciences, Ontario Veterinary College, University of Guelph, Guelph, ON N1G 2W1, Canada; clocking@uoguelph.ca

**Keywords:** PFAS, PFOS, PFOA, fertility, endocrine-disrupting compounds, oocyte, sperm, embryo, ovarian reserve

## Abstract

There has been emerging research linking per- and poly-fluoroalkyl substances (PFAS) to gamete viability and fertility. PFAS, prevalent in the environment and water supplies, undergo slow degradation due to their C-F bond and a long half-life (2.3–8.5 years). In females, PFAS inhibit the hypothalamic–pituitary–gonadal (HPG) axis, reducing follicle-stimulating hormone (FSH) and luteinizing hormone (LH) levels, leading to the inhibition of androgen and estradiol production. PFAS have been found to cause detrimental effects on egg quality through impairing folliculogenesis. In males, PFAS can impair sperm motility and morphology: two fundamental qualities of successful fertilization. PFAS exposure has been proven to inhibit testosterone production, sperm capacitation, and acrosomal reaction. After fertilization, the results of PFAS exposure to embryos have also been investigated, showing reduced development to the blastocyst stage. The aim of this review is to report the main findings in the literature on the impact of PFAS exposure to gamete competency and fertilization capability by highlighting key studies on both male and female fertility. We report that there is significant evidence demonstrating the negative impacts on fertility after PFAS exposure. At high doses, these environmentally abundant and widespread compounds can significantly affect human fertility.

## 1. Introduction

### 1.1. PFOA and PFOS and Their Mechanism of Action

**Figure 1:**
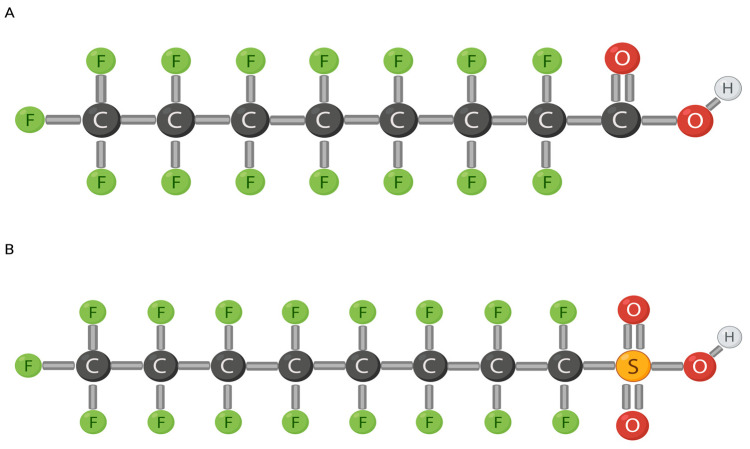
Chemical makeup of PFOS (**A**) and PFOA (**B**). Modified from [5] (**A**) and [6] (**B**). Created with Biorender.com (accessed on 26 January 2024).

Per- and poly-fluoroalkyl substances (PFAS) are a man-made chemical group of non-polymers [1], containing both short-chained (≤7 carbons) and long-chained (≥8 carbons) compounds [2]. The two most studied long-chained PFAS are Perfluorooctanoic Acid (PFOA) and Perfluorooctyl Sulfonate (PFOS), both comprising eight carbons (Figure 1) [2,3,4].

PFAS contain subgroups of perfluoroalkyl acids (PFAAs) including PFOA and PFOS, the most abundant members of PFAS (Figure 1). PFOA and PFOS compounds comprise a fully fluorinated eight-carbon chain along with a functional group at the terminal carbon and, typically, hydrogen or oxygen (or any non-fluorine atom) binds to the other non-fluorinated carbons [1]. PFAS are very persistent in the environment because the C-F (perfluoroalkyl moiety) bond has a high electronegativity with a small fluorine atom, making it very stable [1,2,7]. A strong bond, such as the C-F bond, requires a lot of energy to break. Fluorine has a low polarity which strengthens PFAS hydrophobicity and lipophobicity, that are positively associated with carbon length [8,9]. Increased carbon length also increases neurotoxic effects [10]. Similarly, functional groups contribute to the properties of PFOAs as well. More specifically, carboxyl compounds are easier to degrade due to their high electrophilic properties [1]. A strong distinctive characteristic of PFAS is the ability to encompass both hydrophobic and lipophobic properties. PFAS play a role in lipid metabolism and have been positively associated with serum concentrations of cholesterol [9]. If ionization occurs in aqueous environments, the anion formed will impact environment abundance as PFOS is more resistant than PFOA due to the fluorinated carbon length [1,11]. 

PFAS are extremely resistant to degradation, both environmentally and metabolically. Additionally, PFAS are extremely thermally and chemically stable [1,12]. Primarily, PFAS are excreted by the body through urine, with other routes of elimination being breast milk and menstrual blood [13,14]. Additionally, the body excretes PFAS through pregnancy as PFAS concentrations have been found in newborns and the placenta, leading to negative birth outcomes [15,16]. The high resistance and low elimination rate can be linked to the long half-life of 2.3–8.5 years [7,17]. More specifically, the half-life is 4.8–5.4 years in serum for PFOS and 3.5–3.8 for PFOA [18]. Unfortunately, there has not been much success with environmental elimination treatments for PFAS.

Constant exposure to PFAS in the environment leads to accumulation in the body. Environmental exposure has led to high concentrations found in the blood and lungs due to their amphiphilic properties and high affinity for proteins such as albumin [1,2,19]. PFAS can pass through the blood ovarian follicle barrier and have been detected in follicular fluid, impacting female reproduction [14,20,21,22,23]. Other notable concentrations have been found in serum, seminal fluid, thyroid, reproductive organs, brain, and many fetal-dependent structures, such as the umbilical cord and breast milk [3,17]. PFAS undergo enterohepatic circulation, accumulating in the liver, rather than being stored in adipose tissue, contrary to other organic pollutants [13]. There is a positive correlation between PFAS concentrations and hepatotoxicity as increased PFAS lead to chronic liver disease and decrease hepatic function [24]. 

PFAS are classified as endocrine-disrupting compounds (EDCs). EDCs have agonist characteristics by impersonating endogenous hormones through receptor-mediated disruption [25]. Typically, EDCs act via nuclear hormone receptors to impact the transcription of specific genes, but they can also present with antagonist features by inhibiting ligand–receptor interactions and blocking typical responses [25]. Most commonly, EDCs mimic estrogen and androgen allowing for interference with sexual hormone signaling and weakening reproductive health [17]. Additionally, the aryl hydrocarbon receptor (AhR) is a ligand-activated transcription factor that is a common binding site for EDCs [25]. Being broadly expressed across the body, AhR is commonly activated by xenobiotics. AhR can also indirectly impact hormone signaling by cross-talking with further nuclear receptors [5,25].

Although not confirmed, it is speculated that the nuclear hormone receptor activity is inhibited by PFAS exposure, leading to the disruption of steroid hormone synthesis [5]. PFAS impersonate endogenous ligands, involved in cell signaling when entering the body, most commonly the peroxisome proliferator-activated receptors (PPARs) [5]. PPARs are found in various cell tissues and play a role in cell regulation as ligand-activated transcription factors [5]. Typical ligands for PPARα include eicosanoids and fatty acids [26]. PFAS and other xenobiotics can pose as ligands and activate PPAR complexes, acting as agonists [11,26]. The activation of PPAR complexes can cause unnecessary downstream effects. In reference to hormone production, PPARγ obstructs proper estrogen synthesis through inhibiting the aromatase enzyme, leading to decreased estrogen production and the accumulation of androgen abundance. Changes in hormone levels can cause drastic effects in reproduction [27]. Additionally, the activation of PPARs in hepatocytes causes hepatotoxicity [28]. PFOS is a stronger agonist compared to PFOA [28]. This is due to the presence of sulfonic acid, as it induces irregular spindle morphology through the polymerization of tubulin and is a stronger acid [20,28]. It has also been found that PFAS toxicity increases with carbon length [20]. Additionally, similar results are found in the pancreas. Pancreatic toxicity results in the downregulation of crucial developmental genes, such as SOX9, required to produce key proteins during development [5]. 

Other nuclear pathways of activation include initiating constitutive androstane receptor (CAR) and pregnane X receptor (PXR) [28]. Thyroxine (T4) levels in the thyroid are impacted through the binding between PFAS and the thyroid hormone transport protein, transthyretin [2]. These are just selected examples of several mechanisms through which PFAS affect various regulatory systems in the body.

### 1.2. Toxic Environmental Exposure

Due to the endocrine disruption properties of PFAS, there are many concerns regarding the toxicity of these compounds [1]. PFAS are found in many household items, including ‘anti-stick’ cookware, stain repellent, paint, paper plates, pizza boxes, and cosmetic products [1,4,29]. Water acts as the primary environmental reservoir for PFAS, though concentrations can also be detected in the atmosphere, soil, and animal tissues/food packaging [1,30]. Additionally, there is a growing area of research surrounding the toxic effects on firefighters due to the common use of PFAS in fire extinguishers and fireproofing materials [1].

PFAS have been detected in 100% of tested individuals with the primary source of exposure being drinking water, as PFAS are present in wastewater that is not properly filtered [31], and the second source being diet [32]. 

In 2002, the global producer of PFOS terminated production due to its proven toxic effects [33]. For the most part, Canada has stopped all direct use of PFAS, with PFAS still in fume suppressants and firefighting equipment [33]. However, virtually all Canadians have PFAS concentrations in their bloodstream from environmental exposure, leading the Canadian Ecological Screening Report to conclude that PFOS follows the CEPA 1999 Persistence and Bioaccumulation Regulations in Persistence and recommending that PFOS be added to the List of Toxic Substances by the Government of Canada [33]. This should inhibit the development, use, and importation of such compounds. Looking at long-term exposure to PFOS, carcinogenic effects have been observed but at higher doses than those present in the environment [33]. For example, emerging studies have begun to directly link PFAS exposure to an increased likelihood of developing breast cancer [7].

Concentrations of 20 ng/mL and above are considered toxic levels of PFOS and PFOA [34]. Amounts of 1 to 50 ng/mL of PFAS in serum are the standard levels measured as typical exposure [35]. A potential health risk is observed with concentrations between 2 and 20 ng/mL [34]. However, serum levels as high as 227.6 ng/mL have been found in specific areas, for example, Little Hocking, Ohio, due to water contamination from a nearby manufacturing plant [34]. Health effects are not expected to occur from PFAS exposure lower than 2 ng/mL [34].

The Government of Canada has issued a guide for the adverse effects observed after daily oral exposure to PFAS [36]. Specifically, for reproduction, PFAS levels are measured in milligrams per body weight each day (mg/kg bw/day) [36]. To produce adverse effects in male reproduction, PFAS exposure is determined to be between 0.01 and 500 mg/kg bw/day, while for females it is between 0.2 and 1000 mg/kg bw/day [36]. PFAS affect reproductive hormones production in the range of 0.2 to 200 mg/kg bw/day [36].

PFAS are abundant in the environment, affect 100% of the population, and accumulate readily in the body [31]. High doses are carcinogenic and have been found in the lungs, blood, thyroid, seminal and follicular fluid, male and female reproductive organs, brain, umbilical cord, and breast milk [33]. The amphiphilic properties and high affinity for albumin allow for the circulation of PFAS in the body [30]. The high affinity for abundant serum proteins explains their weak toxicity despite their long half-life. Unfortunately, there has been little success with efforts to eliminate PFAS due to the presence of strong carbon–fluorine bonds. The greatest two sources of exposure, water and diet, cause general exposure rates of 2.0–20.0 ng/mL, with concentrations above 20.0 ng/mL leading to deficits in normal function [34]. PFAS pose an increased risk to human health; and for the purpose of this review, we will be highlighting the effects of PFAS exposure on the gametes and reproductive functions.

## 2. Methods

A literature search was conducted using PubMed, Web of Science, and Google Scholar. We chose these databases to locate accurate and relevant publications. We searched the databases using the following keywords; PFAS, PFOS, PFOA, fertility, endocrine disrupting compounds, oocyte, sperm, embryo, ovarian reserve alone and in combination. Our primary search terms used were ‘PFAS effects on female gametes’ and ‘PFAS effects on male gametes’. We searched for publications up to February 2023 and prioritized more recent publications to ensure that our review has an accurate representation of current data. We applied filters for [2020-present] as well as [English] and examined those selections first. Primarily, we preferred studies performed on human subjects, but since this topic is niche, we accepted any appropriate and significant results. Methods and data were extracted from studies to compare and compile a current assembly of results. 

## 3. Effects on Reproductive Hormones

For the purpose of this chapter, the endocrine-disrupting effects of PFAS exposure will be highlighted. 

### 3.1. GnRH

**Figure 2:**
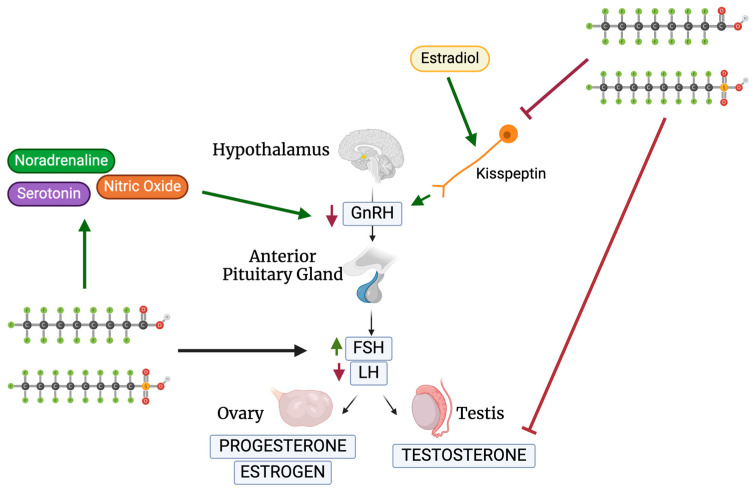
Schematic of PFOS and PFOA’s effects on the HPG axis. Created with Biorender.com (accessed on 2 May 2024).

Gonadotropin-releasing hormone (GnRH) regulates the release of anterior pituitary gonadotropins, luteinizing hormone (LH), and follicle-stimulating hormone (FSH), by acting as the primary hypothalamic hormone [37,38]. LH and FSH induce the gonads to generate sex steroids to begin gamete production [37]. Testosterone (T), estrogen (E2), and progesterone (P4) are the three primary steroids produced (Figure 2).

A study performed by Du et al. looked at the sexual maturation changes in juvenile rats after PFAS exposure, specifically targeting steroid-sensitive kisspeptin (*Kiss1*) (Table 1) [39]. *Kiss1* neurons, regulated by estradiol, control GnRH release and monitor the hypothalamic–pituitary–gonadal (HPG) axis (Figure 2) [40]. Du et al. found that the injection of PFOA or PFOS (0.1, 1, 10 mg/kg) during postnatal days 1–5 (PND1-5) inhibited *Kiss1* and *Kiss1r* mRNA in the anteroventral periventricular nucleus (AVPV) and arcuate nucleus of the hypothalamus (ARC) [39]. Maintaining *Kiss1* levels in the hypothalamus is crucial for proper reproductive development during puberty. During PND26-30, the effects of AVPV *Kiss1* depended on the dose of PFOA as a lower dose of 0.1 mg/kg PFOA increased the expression of AVPV *Kiss1*, in comparison to a higher dose of 10 mg/kg PFOA that decreased *Kiss1* in ARC [39]. Du et al. thus concluded that PFOA majorly impacts the regulation of the HPG and steroid production through the regulation of *Kiss1* [39]. These findings suggest that PFAS act indirectly through neurons as well as directly through GnRH hormones, proving to be a threat to the HPG axis. 

Since some neurotransmitters and hormones, such as noradrenaline, regulate GnRH release, López-Doval et al. wanted to examine if PFAS inhibit the HPG axis through targeting neurotransmitters [41,42]. López-Doval et al. looked at the role of noradrenaline, serotonin, and nitric oxide on rat reproductive disruption [42]. These three signals stimulate GnRH neurons and regulate GnRH release. PFOS exposure in adults significantly increased serotonin concentrations in the hypothalamus directly proportionally to the increase in PFOS concentrations [42]. Similarly, neuropeptide Y expression was decreased when concentrations of above 1 mg/kg/day were administered, while nitric oxide signaling genes were amplified above 3.0 mg/kg/day [42]. There was an increase in noradrenaline concentrations in the anterior hypothalamus—where GnRH and *Kiss1* are stimulated [42]. Minimal changes in signal expression can impact the balance of the HPG axis as PFOS effects on reproductive signal regulation, through serotonin and neuropeptide Y, suggest HPG dysfunction and GnRH inhibition [42]. A sequential study conducted by the same research group further investigated the exact mechanism of PFOS on GnRH release. PFOS was administered at 1.0, 3.0, and 6.0 mg/kg/day for 28 days to adult male rats (Table 1) [43]. The GnRH receptor protein was inhibited at all three doses while the pituitary GnRH receptor was left unchanged. PFOS administration in adult rats impairs the GnRH signaling release through the protein receptor, *Kiss1* expression, and pituitary gonadotropin production, which impairs the HPG axis [43]. Surprisingly, in testes, PFOS stimulated LH receptor gene expression. These results demonstrate the varying mechanisms through which PFAS act, both directly and indirectly, on the HPG axis. 

Austin et al. looked at the neuroendocrine effects after PFOS injections in adult female rats (Table 1) [44]. The rats were injected with PFOS for 2 weeks at 0, 1.0, and 10 mg/kg, resulting in PFOS detection in the brain tissue. Similar to the study conducted by López-Doval et al., there was induced norepinephrine in the hypothalamus suggesting that estrous cycle disruption was the result of an affected hypothalamic–pituitary–adrenal (HPA) axis [42]. There was inhibition of regular estrous cyclicity in adult females after exposure as the 1.0 mg/kg group had only 66% regular cycles with a further decrease to 42% in the 10 mg/kg group [44]. Additionally, there was an increase in persistent diestrus from 8% to 33%, reducing the fertility window. These results were particularly interesting as concentrations of PFAS were found in the brain tissue. This could show neurological effects on the body outside of the scope of reproduction. More research needs to be conducted in this area. 

Overall, these studies show a consistent inhibition of GnRH from PFAS exposure. Indirectly, PFAS can inhibit HPG signaling through neurons such as *Kiss1* as well as hormones and neurotransmitters. Directly, PFAS inhibit GnRH concentrations which further alter FSH and LH levels. They were found to have a stronger impact on the hypothalamus than the pituitary gland but nonetheless shifted the balance of the HPG axis, which is very crucial to regulating the reproductive system. 

### 3.2. FSH and LH

LH is required for androgen synthesis, estradiol synthesis, and the initiation of follicular growth [45]. LH activates the theca cells inducing the conversion of P4 to androstenedione (A4) and activating aromatase, which converts A4 to E2 (Figure 2) [45]. FSH controls follicular growth and estrogen synthesis as well as works alongside LH to activate aromatase [45]. Decreased GnRH will further decrease the activation of LH and FSH. A reduced concentration of LH leads to a decreased or delayed ovum release, majorly affecting fertility by shortening the window for fertilization to occur. If the signal to stimulate the development of the ovum is below the required threshold, follicular development will not occur [45].

Wang et al. looked at the effects of PFOS exposure on reproductive ability through estrogen receptor ⍺-activated kisspeptin neurons in female mice (Table 1) [46]. In the 10 mg/kg group, an overall increase in diestrus and decrease in corpus luteum were noted, further decreasing ovulation. They showed that LH and FSH decreased after PFOS exposure during proestrus and LH was elevated during diestrus. However, FSH remained at low levels while there was an overall decrease in AVPV *Kiss1* expression, similar to the study performed by Du et al. [39,46]. E2 concentrations were also reduced alluding to a decrease in AVPV kisspeptin neurons required for an LH surge through the disruption of the E2-modulated pathway [46]. LH surge during proestrus was compromised after exposure to PFOS; therefore, the exposure to high doses of PFOS negatively impacts the neurons controlling the HPG, by targeting genes and proteins. Once treated with kisspeptin agonists, the diestrus cycle was repaired and the reduction in the corpus luteum stopped. The importance of these data shows that in different species, PFOS impacts the HPG axis the same way. Clearly, *Kiss1* is a strong target of PFOS which is detrimental to our reproductive system. 

### 3.3. Testosterone Levels in Females

**Figure 3:**
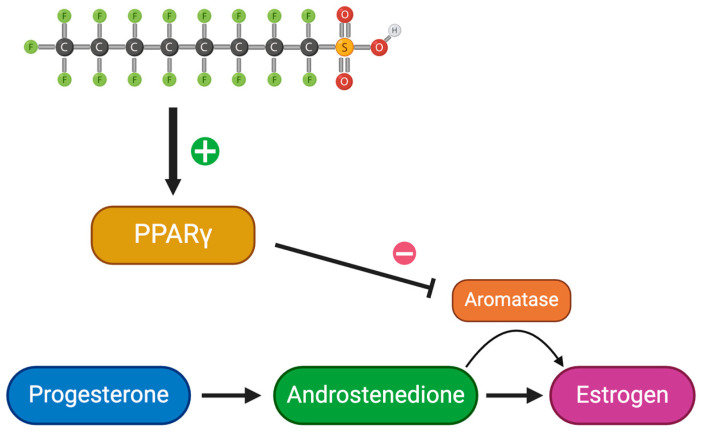
Schematic of estrogen synthesis impaired by PFAS. Created with Biorender.com (accessed on 25 April 2024).

Peroxisome proliferating activating receptor gamma (PPARγ) isoforms are targeted receptors to PFAS (Figure 3) [27]. Chaparro-Ortega et al. collected ovaries and examined granulosa and theca cells after PFOA treatment (Table 1) [47]. While looking at PFAS’ effects on porcine ovarian cell steroidogenesis, PFOA had a strong effect on granulosa cells by activating PPARγ. On the other hand, PFOS had a strong effect on theca cells leading to the activation of PPARα and PPARβ/δ [47]. This can be explained by understanding the different PPARs that are expressed in theca and granulosa cells. In theca cells, PFOS concentrations of 1.2, 12, and 120 µM significantly increased P4 secretion, while 0.12, 1.2, and 12 µM decreased A4 secretion [47]. Looking at granulosa cells, a concentration as low as 0.12 µM decreased P4 and E2 secretion. This demonstrates the ability of PFAS to target different cell types using various receptors. Outside the area of reproduction, there may be other strong effects as a result of these activated receptors. More research needs to be conducted to further evaluate this. Due to the gonadotropic stimulus with follicle growth, PPARγ activation increases [47]. PPARγ inhibits aromatase, leading to a potential decrease in estrogen alongside an increase in androgens—one being testosterone [27]. Elevated testosterone levels in women are a common sign of hyperandrogenism and polycystic ovary syndrome (PCOS) [27,32,48]. Additionally, women with elevated testosterone may demonstrate symptoms similar to those of PCOS, such as larger antral follicles [27,32,48]. Testosterone is a major contributor to the production of estradiol and optimal testosterone levels are crucial for healthy ovarian function, as an excess or depletion of testosterone can throw off the balance [27,48]. Increased testosterone, caused by PFAS’ inhibition of aromatase, can impact follicle cycle and development by stopping it at the antral stage [22,48]. A prolonged follicular phase and delay in ovum release can lead to decreased fertility by shortening the woman’s ovulation window. Additionally, affecting follicular development can lead to declined ovulation rates, oocyte development, and an overall decline in follicle health [21,23,48]. A decrease in overall progesterone levels can help explain abnormal ovulation. Androgen excess, specifically testosterone, can result in impaired zygote development with a smaller percentage reaching the blastocyst stage [48]. In other words, increased levels of testosterone lead to oocyte incompetence.

### 3.4. Cyp17 Gene Expression

Cytochrome P450 (CYP) genes synthesize and metabolize estrogen and xenobiotics [7]. Cytochrome P450 17-alpha-hydroxylase/C(17, 20)-lyase (CYP17) participates in estrogen biosynthesis by transforming the precursors of androgens and estrogens from pregnenolone and progesterone [7]. It demonstrates both hydroxylase and lyase activity. Inhibition of the CYP17 enzyme by activating PPARγ decreases P4 conversion to testosterone in ovarian follicles [47]. This suggests that the damage to the P4 to A4 conversion is caused by PFOS. A4 plays a crucial role in producing testosterone and estrogen, further affecting ovulation and sperm production. Additionally, Cyp17a1 plays a vital role in spermatogenesis and overall fertility in males through the production of testosterone and 11-ketotestosterone (11-KT) [7,49,50]. Yang et al. studied Cyp17a1 in zebrafish to observe the implications of different genotypes of the Cyp17a1 gene (Table 1) [50]. Compared to the fish with the cyp17a1 gene (cyp17a1 +/+), thinner efferent ducts and degeneration of the gonads were analyzed in cyp17a1−/− subjects [50]. Additionally, there was a major decrease in spermatogenic cell count in the testes of the cypa1 −/− subjects [50]. This highlights the important role that Cyp17a1 plays in fertility as Cyp17a1−/− subjects’ continued deficiency resulted in sterility [50]. Therefore, PFOS’s activation of PPARγ leading to the inhibition of the CYP17 gene ultimately decreases fertility and leads to detrimental results. 

**Table 1:** Summary of findings of PFAS’ effects on reproductive hormones.

Species	In Vivo/In Vitro	Cell Type	Endpoint	Treatment Dose (mg/kg)	Effect Observed	Reference
*Rattus rattus*	in vivo	n/a	Detect a change in sexual maturation, looking at Kiss1, after PFAS exposure	0.1, 1.0, and 10 mg/kg of PFOA or PFOS during PND1-5	- 0.1 mg/kg: increased expression of Kiss1 - 10 mg/kg: decreased expression of Kiss1	[39]
*Rattus rattus*	in vivo	n/a	Effects of PFOS exposure in GnRH	0.5, 1.0, 3.0, 6.0 mg/kg/day for 28 days	- Decreased GnRH expression at 0.5, 1.0, and 3.0 mg/kg - Increased noradrenaline - GnRH receptor protein was inhibited at all doses	[43]
*Rattus rattus*	in vivo	n/a	Neuroendocrine effects of PFOS in regard to the estrous cycle	1 and 10 mg/kg of PFOS for 2 weeks	- Induced norepinephrine in hypothalamus - Inhibited regular estrous cyclicity to 66% at 1 mg/kg and 42% at 10 mg/kg - Increased diestrus persistence from 8% to 33%	[44]
*Mus musculus*	in vivo	n/a	PFOS exposure effects on ovulation stages of mice	10 mg/kg of PFOS for 14 days	- PFOS exposure decreased LH and FSH - LH elevated during diestrus - E2 and Kiss1 decreased	[46]
*Sus scrofa*	in vitro	Porcine theca and granulosa cells	PFAS’ effects on porcine ovarian cell steroidogenesis	0.12, 1.2, 12, and 120 µM of PFOS and PFOA	PFOA: - PPARγ affects granulosa cells, −0.12 decreased P4 and E2 PFOS: - PPARα and PPARβ/δ affect theca cells - 1.2 µM+ increased P4 and decreased A4	[47]
*Danio Rerio*	in vivo	n/a	Observe the implications of different genotypes of the Cyp17a1 gene	n/a	- Cyp17a1−/−: thinner efferent ducts and gonad degeneration - Decrease in spermatogenic cell count and sterility	[50]

## 4. Effects on Female Gamete Development

For the purpose of the following chapters, the direct reprotoxic effects of PFAS exposure will be highlighted. 

### 4.1. Oocyte Competence

The ovary undergoes two essential processes: folliculogenesis and steroidogenesis [13]. Oocyte maturation significantly depends on gap junction intercellular communication (GJIC) between the oocyte and granulosa cells [51]. To analyze the effects of PFOA on GJIC, López et al. investigated the oocyte–cumulus cell communication (Table 2) [51]. After exposing one group of mice to 2.5 mg/kg/daily PFOA for 3 days, cumulus–oocyte complexes (COCs) were removed from the ovaries and analyzed in a GJIC assay using calcein [51]. High fluorescence in the DMSO control group and little to none in the PFOA-treated COCs were detected, suggesting that PFOA causes a GJIC obstruction in COCs as well as diminished levels of factors and molecules essential for oocyte growth, leading to oocyte death [51]. Additionally, López-Arellano et al. further studied PFOA’s in vitro effects on mouse oocytes using concentrations of 50, 100, and 150 μM (Table 3) [51]. While 50 μM PFOA did not change oocyte viability, 150 μM PFOA resulted in the death of all oocytes exposed. To observe the apoptotic and necrotic effects in PFOA-exposed oocytes, López et al. subjected different samples to varying PFOA concentrations, between the range of 28.2 and 112.8 μM PFOA, with 112.8 µM representing the LC_50_ of PFOA and 28.2 μM being the occupational exposure dose of PFOA in the environment [51]. After 24 h of exposure, there was a significant increase in apoptotic and necrotic oocytes compared to the DMSO control group. A positive correlation between PFOA concentration and apoptosis was established. PFOA interference with essential GJIC leads to inhibited oocyte development. 

Mitochondria are the biggest producer of reactive oxygen species (ROS) as they regulate energy through oxidative phosphorylation [52]. ROS are a group of oxygen-containing species that are highly reactive and are a normal by-product of metabolism and cellular processes [51]. Antioxidant systems of the cell control the concentration of ROS and protect the cell from ROS injury [51]. If the defense system fails, ROS can cause a lot of damage to cell structures and can lead to disease. Excess ROS and activated PPARs results in DNA damage, protein degradation, and lipid peroxidation, which leads to oocyte death [13,17,51]. Additionally, PFAS bind to PPARγ and impair the genes involved in meiosis [13]. Increased ROS abundance suggests strong mitochondrial dysfunction [52]. 

PFOA directly impacts energy production as Zhang et al. found a significant decrease in the ATP content while looking at mitochondrial metabolic effects during oocyte maturation (Table 2) [52]. They found that the ROS fluorescence intensity increased as PFOA doses increased, from 1.0 mg/kg to 4.0 mg/kg. An increase in PFOA exposure decreases ATP and DNA production through mitochondrial damage in the oocytes, greatly affecting the stability and viability of oocytes [52]. Using the same concentrations as in their previous experiment, López et al. also tested the effect of PFOA on ROS levels in ovaries (Table 2) [51]. Controlled and PFOA-exposed ovaries were cryostat sectioned and DCF was added to detect ROS presence [51]. There was a significant increase in fluorescence in both groups of PFOA ovaries compared to the negative control. Additionally, the 112.8 μM sample had the same fluorescence as the positive control (H_2_O_2_), suggesting a strong ROS increase following PFOA exposure [51]. High concentrations of ROS can be very detrimental to developing cells, particularly gametes. Elevated ROS levels lead to mitochondrial dysfunction and can inhibit growth. It is crucial to develop viable oocytes and any danger to viability is very damaging. 

Connexin (Cx) hexamers form gap junctions in mammalian ovaries and regulate folliculogenesis [53,54]. In mice, Cx43 is the major moderator of granulosa–granulosa cell communication. High levels of guanosine monophosphate (cGMP) and cyclic adenosine monophosphate (cAMP) maintain the oocyte in a meiotic arrest. Before ovulation, a gonadotropin surge occurs to stimulate the oocytes to resume meiosis rupturing the GJIC as a broken GJIC results in a decrease in cAMP and cGMP leading to meiotic growth [53]. Connexins also regulate signal transduction, and control developing tissue growth [54]. There was a positive correlation between the concentration of PFOS and the number of viable oocytes after exposure [53]. Domínguez et al. exposed COCs to 12.5, 25, and 50 µM of PFOS to examine GJIC communication in porcine oocytes (Table 3) [53]. Of the 210 oocytes examined, exposure to a concentration of 25 μM led to only 126 viable oocytes, a significantly lower number than the control, resulting in a 40% decrease in live oocytes. Additionally, an exposure to 50 μM PFOS led to 4 viable oocytes from an initial 210, a 98% decrease, strongly supporting the lethal toxicity of 50 µM. Cx43 serves as an oocyte quality marker and, if targeted by PFAS, can be detrimental to oocyte viability [54]. Moreover, looking at oocyte maturation after PFOS exposure, there is a positive correlation between PFOS concentration and the number of mature oocytes, with a significant difference in the maturation levels between the control and the 50 μM PFOS group [53]. In the control, there were 13 oocytes in the germinal vesicle stage (GV), 32 in metaphase I (MI), and 67 in metaphase II (MII), while in the 50 μM PFOS exposure group, there was a significant change in the oocytes in GV and MII with 67 GV, 26 MI, and 5 MII, respectively. This demonstrates that an increasing concentration of PFOS negatively affects oocyte maturation and can impact viability, leading to a potential decline in fertile gametes. 

Overall, these studies show that PFAS massively damage oocyte viability. PFOA prevents essential cell communication precluding oocytes from proper developmental nutrients. Additionally, PFOA causes mitochondrial dysfunction by increasing ROS. Increased ROS also has detrimental effects on nuclear function and can cause severe genetic damage. Finally, PFOS directly destroys viable oocytes when concentrations become too high. PFAS exposure leads to negative outcomes that will greatly affect fertility. 

### 4.2. Oocyte Reserve

An Ovarian Sensitivity Index (OSI) measures ovarian competence to FSH stimulation [55]. OSI measurements are a strong indication of pregnancy rate and are an accurate biomarker of Assisted Reproductive Technology (ART) success and female fertility [55]. While studying the link between chemical exposure and female fertility, Bellavia et al. proved that higher concentrations of PFAS resulted in a lower OSI measurement through ovarian sensitivity interference in women [55]. However, in a study looking at pollutant levels in fluid, conducted by Björvang et al., conflicting results showed that PFAS exposure was not found to impact the OSI measure but instead affected embryo quality [5]. Similarly, Feng et al. looked at the effects of PFAS carbon-chain length (Table 3) [20]. COCs collected from mice were exposed to 600 µM of PFOS and PFOA. As the carbon-chain length of PFOS compounds increased beyond eight, there was a lower germinal vesicle breakdown (GVBD) and a lower polar body extrusion (PBE) rate, confirming the toxicity associated with increased carbon-chain length. The addition of a sulfonate group also significantly increases the PFOS toxicity compared to PFOA [20,28]. Cleavage rates of PFOS-exposed oocytes were significantly lower than control rates, specifically 70.4% and 62.2% lower than controls [20]. Lower GVBD and PBE rates suggest the high toxicity of PFAS. Additionally, decreased cleavage rates can be detrimental to proper development and are partly the result of diminished oocyte competence. 

Feng et al. looked at PFOS exposure in relation to suppressed estrogen synthesis in mice, showing that treating mice with 0.1 mg/kg/day PFOS for 6 months and inhibiting the histone acetylation of steroidogenic promoters negatively impacted the ovarian follicular reserve through the inhibition of ovarian hormones and decreased follicular development (Table 2) [56]. This demonstrates that PFOS exposure causes decreased follicle maturation through the reduced synthesis of E2. PFAS exposure decreases total corpora lutea and can lead to follicular atresia [13]. Zhang et al. led a study looking at the effects of 28 days of PFOA exposure on the estrous cycle (Table 2) [52]. The low-dosage, 1 mg/kg/day, PFOA exposure group had a significantly shortened estrus phase, while the high-dosage group, 5 mg/kg/day, had significantly extended metestrus and anestrus phases. A fertility test conducted on the mice showed that both exposed groups had a significantly smaller litter size [52]. Similarly, mice in both PFOA-exposed groups had smaller ovaries compared to the control group, when measuring ovary weight and size. Additionally, there was a significant decrease in the number of primordial follicles in the low-dose group, suggesting a decrease in the ovarian reserve. More specifically, there was an overall significant decrease in the number of follicles in all stages for the high-dose PFOA-exposed group and a significant decrease in the total ovarian follicle count in both the low- and high-dose groups. Henceforth, there is a negative correlation between ovulated oocytes and PFOA dosage. The mechanism by which PFOA impaired mature oocyte development is through undergoing symmetrical division for release of the polar body [52]. Typically, this division is asymmetrical, which allows for a smaller polar body (PB) and larger secondary oocyte. A more symmetrical division creates abnormally sized cells. The GVBD rate was significantly reduced 3 h after culture of both the low- and high-dose groups and a portion of oocytes in the low-dose PFOA-exposed group were not able to resume meiosis [52]. 

Overall, there are many consistent conclusions between studies. PFAS compounds target PBE and impact crucial steps in meiosis. Symmetrical division of the polar body leads to a smaller oocyte remaining and can massively impact the viability of the oocyte as it finishes meiosis, in the worst cases, leading to oocyte death. Any changes to meiosis or cleavage rates create detrimental effects that may prevent fertilization. This could also be a result of smaller ovaries’ development after exposure. Additionally, prior to meiosis, after PFOS exposure, there is a decrease in follicle maturation and an overall decrease in follicle numbers, further reducing ovarian reserve. As a result, smaller litter sizes were noted in mice [52]. 

### 4.3. Chromosome Misalignment, F-Actin Organization, and Spindle Formation

First PB abnormalities are related to poor outcomes in in vitro fertilization (IVF) and are typically used to determine egg quality [20]. Abnormalities such as fragmentation and enlargement are some of the key observational features. The cleavage plane is determined by the position of the spindle as, during meiosis I, it migrates towards the cortex. This is regulated by F-actin and allows the small PB to be expelled, while maintaining in the oocyte all RNA and proteins necessary for fertilization [20]. Feng et al. investigated the effects of the PFAS carbon-chain length and found an increased sized PB after exposure to PFAS, suggesting a cytoskeleton organization interruption in the oocyte [20]. These results coincide with the symmetrical division found after PFOS exposure, impacting the continuation of meiosis due to oocyte size [52]. To confirm this hypothesis, they stained the chromosomes, microtubules, and F-actin for PFOS. Abnormal F-actin abundance was found in the cytoplasm, suggesting a decreased migration of the spindle to the cortex as well as a decreased F-actin cage. Spindle migration increased significantly in PFOS-exposed oocytes compared to controls. There was also an increase in the length–width and length–diameter of the PB after exposure resulting from migration failure [20]. Elongated spindles were observed in the exposed groups, and it was proposed that the oocyte was compensating to produce a normal-sized PB [20]. The compensation of the oocyte demonstrates that, although PFOS affects the oocyte during meiosis, significant effects may not be recognized until fertilization. Additionally, it was noticed in oocytes missing MAP kinase activity (mos−/−) that elongated spindles resulted in allowing one pole to be close to the cortex, while the other was near the center. The mos−/− oocyte produced normal-sized PBs, similar to those of the PFAS-exposed groups. Although metaphase II can be reached in these oocytes, fertilization is majorly compromised due to incorrect development [20], further proving that PFOS greatly impacts meiosis leading to decreased oocyte developmental success. Without the proper development of gametes, fertilization cannot occur.

**Table 2:** Summary of findings of in vivo PFAS’ effects on oocyte competence.

Species	Endpoint	Treatment Dose	Effect Observed	Reference
*Mus musculus*	PFOA effects on ROS levels in the ovary ex vivo	28.2 and 112.8 μM of PFOA for 24 h	- Significant increase in fluorescence - 112.8 μM: same fluorescence as positive control	[51]
	PFOA effects on GJIC	2.5 mg/kg/daily for 3 days	- DMSO: high fluorescence - PFOA-COC: no fluorescence	
*Mus musculus*	Impact of PFOS exposure on ovarian hormone production	0.1 mg/kg for 6 months	- Inhibited ovarian hormones and follicular development	[56]
*Mus musculus*	Exposure to PFOA’s impact on the estrous cycle	Low dose, 1 mg/kg/d, and high dose, 5 mg/kg/d, of PFOA for 28 days	Low-dose group: - Significantly shorter estrus phase - Significant decrease in primordial follicles High dose: - Significantly extended metestrus and anestrus phases - Significant decrease in total ovarian follicle count Both groups: - Significantly smaller litter groups and ovaries	[52]
	Unstable follicular ovulation and development following PFOA exposure	Low dose, 1 mg/kg/d, and high dose, 5 mg/kg/d, of PFOA for 28 days	- GVBD rate significantly reduced after 3 h - Oocytes in low-dose group could not resume meiosis	

**Table 3:** Summary of findings of in vitro PFAS’ effects on oocyte competence.

Species	Cell Type	Endpoint	Treatment Dose (µM)	Effect Observed	Reference
*Mus musculus*	Denuded murine oocytes	PFOA effects on mouse oocytes	50, 100, and 150 μM of PFOA for 24 h	- 50 μM: no change - 150 μM: death of all oocytes	[51]
		Observe apoptotic and necrotic effects in PFOA exposed oocytes	28.2 and 112.8 μM of PFOA for 24 h	- Significant increase in apoptotic and necrotic oocytes in both exposed groups	
*Sus scrofa*	Porcine oocytes	Effects of PFOS on the number of live oocytes after exposure	12.5, 25, and 50 μM of PFOS of 44 h	From an initial 210 oocytes examined: - 25 μM: significant drop to 126 live oocytes - 50 μM: significant drop to 4 live oocytes	[53]
		Effects of PFOS on the number of mature oocytes after exposure	12.5, 25, and 50 μM of PFOS for 44 h	Control: 13 GV, 21 MI, and 67 MII 50 μM: 67 GV, 26 MI, and 5 MII	
*Mus musculus*	Murine oocytes	Impact of PFAS exposure on embryo quality	600 μM of PFOS and 600 μM of PFOA	- Lower GVBD and PBE rate - Cleavage rates of PFOS exposed oocytes were significantly lower than control rates (62.2%)	[20]
		PFAS exposure effects on oocyte maturation	600 μM PFOS	- Increased PB size - Significant increase in spindle migration	

## 5. Effects on Male Gamete and Fertilization Capability

### 5.1. Sperm Viability

The blood–testis barrier is crossed by PFAS, allowing for direct interaction with sperm [18,57,58]. PFOS and PFOA exposure can impact semen quality by coiling sperm tails, impacting the crucial component of sperm motility through the female reproductive tract [12]. Studies point to decreased sex hormone-binding globulin (SHBG), FSH, and testosterone after increased exposure levels to PFOA and PFOS [12,31]. Additionally, Louis et al. detected a significant increase in bicephalic and immature sperm after PFOS exposure [59]. Environmental exposure to PFAS can have a great effect on fertility if it affects sperm morphology since sperm structure is crucial to function and fertilization capability. 

Leydig cell hyperplasia, commonly found in infertile men, can occur as a result of PFAS exposure resulting in lower testosterone levels [31]. The stimulation by LH of the AC-PKA-CREB-StAR leads to increased cholesterol entry in the mitochondria and hence to increased testosterone synthesis by Leydig cells. This pathway, along with the differentiation of stem Leydig cells, is inhibited by PFOS. Moreover, PFOS and PFOA increase ROS in sperm cells similarly to their effects in the ovum [60]. Interestingly, a mild ROS level helps activate tyrosine protein phosphorylation which in turn induces capacitation. However, a high ROS level is very toxic to sperm cells as generating oxidative stress triggers lipid peroxidation, dysfunction of the mitochondria, and DNA damage, all of which are harmful to the male reproductive system [58,60]. 

Many animal studies have been conducted to analyze the effects of PFAS. Leydig cell hyperplasia and adenomas were the result of PFOS exposure in rats in a study conducted by Zhao et al. (Table 4) [61]. They investigated PFOS effects on testosterone production in rats using 5 and 20 mg/kg [61]. This led to testosterone decline. In the in vivo study, inhibition of 3beta-hydroxysteroid dehydrogenase and 17beta-hydroxysteroid dehydrogenase-3 was detected following PFAS exposure. These two enzymes are crucial for testosterone production; therefore, testosterone levels were significantly lower in the 20 mg/kg group [61]. Decreased testosterone levels in males relate to a number of fertility issues and can lead to malfunctioning sperm. PFAS prove a major threat to male fertility. Many animal studies found that the disturbance of seminiferous tubules, decreased sperm numbers, and decreased motility resulted from PFOA exposure along with increased abundance in the testis and epididymis [57]. Additionally, due to this disturbance, testosterone and progesterone levels are significantly decreased. Androgen-binding protein is produced in Sertoli cells after activation by FSH [62]. Sertoli cells are responsible for the blood–testis barrier, held together by actin and microtube cytoskeleton. Typically, the morphological dysfunction of Sertoli cells occurs following exposure to PFAS, as PFOS destroys the cytoskeleton and disrupts the blood–testis barrier [62].

Testicular testosterone is produced in Leydig cells and PFAS can significantly inhibit steroidogenic enzyme activity in Leydig cells due to their effects on androgen secretion [63]. Additionally, PFAS activate the expression of steroidogenic enzymes. PFOA and PFOS have been found to decrease progesterone, the steroidogenic acute regulatory protein (STARD1), and CYP11A1 [64]. STARD1 works in the mitochondria and controls cholesterol, while CYP11A1 converts cholesterol to pregnenolone [49,64]. Mao et al. used Ethane dimethane sulfonate (EDS) to allow for a puberty development model to be promoted in rats (Table 4) [65]. Amounts of 0, 5, and 10 mg/kg of PFOS were used in in vivo rat models to determine the effect of Leydig cell regeneration, showing that, after PFOS exposure, stem Leydig cell (SLC) proliferation was inhibited, further decreasing Leydig cell (LC) numbers [65]. CYP11A1 presence, a biomarker for the identification of LCs, reveals whether LCs were eliminated. After day 7, no CYP11A1 LCs were detected. Moreover, 35 days post-EDS addition, half of the normal testosterone levels returned in the control group [65]. Full testosterone was detected as a sign of LC regeneration on day 56 with no testosterone detected in the 10 mg/kg PFOS group at day 35. This showed a significant decrease compared to the control. Additionally, both PFOS-exposed groups maintained significantly low testosterone levels at day 56, allowing for the conclusion that PFOS inhibits LC regeneration and proliferation [65]. To further confirm these results, CYP11A1 protein density was reduced at 5 and 10 mg/kg PFOS exposure on day 56 and, similarly, 11B-HSD1 protein density was reduced at 10 mg/kg PFOS exposure on both days 35 and 56 [65]. LC depletion can be very dangerous to the male reproductive system as this further impacts the production of testosterone. Low levels of testosterone lead to numerous male fertility issues. Testosterone biosynthesis occurs in Leydig cells and relies on numerous proteins. One crucial protein is the steroidogenic acute regulatory protein (StAR/STARD1), allowing for cholesterol transfer to the inner mitochondrial membrane [66]. Testosterone biosynthesis is stimulated in Leydig cells by StAR and STARD5, a cytosolic sterol transporter. StAR expression is regulated by the coactivator cyclic AMP-response element-binding protein/regulated transcription coactivators CREB/CRTC2 [66]. PFOS exposure has been found to enhance the phosphorylation of CREB to produce neurotoxic effects [66].

Qiu et al. conducted a study where dosages of 0.5, 5, and 10 mg/kg of PFOS were given to mice to determine testosterone biosynthesis hindrance (Table 4) [66]. Testosterone biosynthesis was measured by assessing the mRNA expression of StAR. After 4 weeks, at dosages of 5 and 10 mg/kg, there was a significant decrease in the presence of StAR mRNA compared to the control. Moreover, the 5 and 10 mg/kg groups showed a significant decrease in sperm count in the epididymis [66]. Exposure to PFOS was found to significantly decrease testosterone levels in the testes of the 5 and 10 mg/kg groups; additionally, germ cell degeneration was detected in the 10 mg/kg PFOS-exposed group [66]. To confirm these findings, Qiu et al. measured in vitro testosterone secretion and StAR mRNA by exposing LCs to 15 and 30 µM of PFOS and showed a significant decrease in testosterone secretion for both PFOS groups (15 and 30 μM) (Table 4) [66]. Furthermore, mRNA expression of StAR in LCs was significantly decreased at both concentrations [66]. Therefore, PFOS exposure causes a significant decrease in testosterone biosynthesis in the testes. 

Similar results are seen with both animal and human studies. Looking at human analysis of the semen quality of 105 patients, Joensen et al. found that high PFAS exposure significantly decreased the average number of normal spermatozoa compared to the low-exposure group (Table 4) [67]. Joensen et al. used a quartile score to separate the participants in PFAS exposure groups: low group: 2–3, intermediate 4–6, high group: 7–8. The high-PFAS-exposed group had 6.2 million normal spermatozoa, while the low groups presented with 15.5 million normal spermatozoa, a significant decrease [67]. This confirms that exposure to high PFAS levels decreases spermatozoa quality. Although a lower sperm count is not as detrimental as a low oocyte number in women, it is still a crucial part of healthy fertility rates. Male sperm count naturally declines with age, but following PFAS exposure, this decline can occur much earlier. 

Clearly there is a consistency with PFAS exposure, decreased testosterone biosynthesis, and sperm count as this is present in both human and animal studies. These results pose big threats to male infertility and can lead to serious issues. As male infertility is more known and researched, further studies have been conducted on the negative impacts of EDCs. Clearly, PFAS are very toxic to sperm development. 

**Table 4:** Summary of findings of PFAS’ effects on sperm quality and development competence.

Species	In Vivo/In Vitro	Cell Type	Endpoint	Treatment Dose	Effect Observed	Reference
Animal Studies						
*Rattus rattus*	in vivo	n/a	PFOS and PFOA exposure effects in Leydig cells	0, 5, and 20 mg/kg PFOS	- Leydig cell hyperplasia and adenomas resulted - Decreased testosterone resulted by inhibition of 3B-HSDH and 17BHSDH3	[61]
*Rattus rattus*	in vivo	n/a	Effect of Leydig cell regeneration after PFOS exposure	0.5, 10 mg/kg of PFOS for 56 days	35 days post: no testosterone levels - Significant decrease Both groups: significantly low testosterone levels day 56 - Inhibited LC regeneration and proliferation - CYP11A1 was decreased at 5 and 10 mg/kg day 56 and 11B-HSD1 reduced at 10 mg/kg for both days 35 and 56	[65]
*Mus musculus*	in vivo	n/a	Determine testicular effects of PFOS exposure	0.5, 5, and 10 mg/kg of PFOS for 4 weeks	- Significant decrease in mRNA presence of StAR - 5 and 10 mg/kg: significant decrease in sperm count and testosterone levels - 10 mg/kg: Germ cell degeneration	[66]
	in vitro	Murine Leydig cells	Proving testicular effects occur after PFOS exposure	0, 15, and 30 μM PFOS	- 15 and 30 mg/kg: Significant decrease in testosterone and mRNA StAR	
Human Studies						
*Homo sapiens*	in vitro	Human spermatozoa	PFAS exposure impairs spermatozoa quality	Quartile score: Low group: 2–3 Intermediate: 4–6 High group: 7–8	- High: significantly decreased average number of normal spermatozoa - High: 6.2 million normal -Low: 15.5 million	[67]

### 5.2. Capacitation Reaction

**Figure 4:**
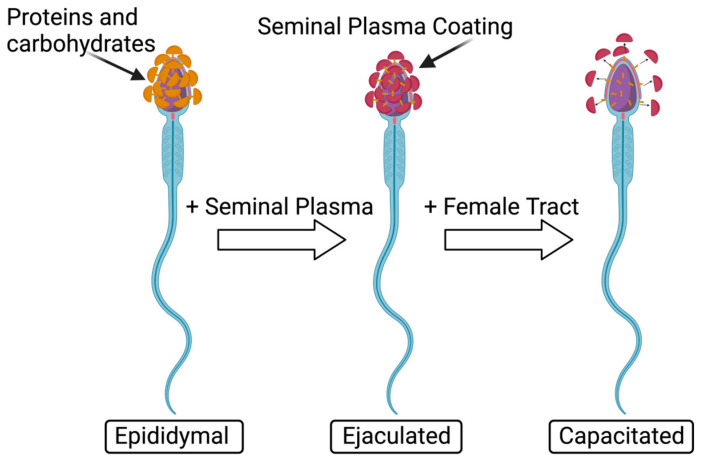
Normal capacitation reaction of sperm. Modified from [69]. Created with Biorender.com (accessed on 29 November 2023).

The capacitation reaction involves the spermatozoa changes, both physiological and chemical, that occur in the oviduct after ejaculation, preparing the sperm to undergo the acrosomal reaction by releasing lytic enzymes responsible for sperm penetration through the zona pellucida (ZP) and exposing membrane receptors on the sperm head, needed to bind to the ZP (Figure 4) [18,68]. 

Sperm capacitation includes an outflow of cholesterol and increased cAMP, which in turn activates protein kinase A, altered membrane permeability, and protein tyrosine phosphorylation [60]. The capacitation reaction increases calcium and bicarbonate ions which are needed to activate the adenylate cyclase, thus resulting in hyperactivated motility [18]. Ca^2+^ increases cAMP which activates protein kinase A pathways and tyrosine phosphorylation, ultimately leading to capacitation [60]. In animal studies, Ortiz-Sánchez et al. investigated PFAS’ effects on plasma membrane dysfunction in boar (Table 5) [18]. They incubated spermatozoa in capacitation conditions of 1000, 1500, and 3000 µM of PFOS and 500, 1000, 1500, 2000, and 2500 µM of PFOA and measured boar sperm cell mortality after exposure to PFOS, detecting it at 64% at 1000 μM and 97% at 3000 μM. After PFOA exposure, mortality levels were 36% at 1500 μM and 76.5% at 2500 μM. These data allow for the conclusion that PFOS is more toxic to sperm cells than PFOA as exposure at lower dosages causes more damage. The mean inhibitory concentration of capacitation (ICC50) of PFOS is 274 μM, meanwhile, that of PFOA is 1458 μM [18]. Looking at just PFOS effects, there was a significant 20% increase in mortality after immediate incubation (0 h) and this increased further to 33% at 4 h [18]. This concludes that PFOS exposure is highly toxic as it demonstrated significant results immediately after exposure. Capacitated patterns, assessed by a Chlortetracycline Fluorescence (CTC) assay, demonstrated that compared to the control spermatozoa capacitation levels of 64%, there was a significant inhibition of capacitation after exposure to PFAS, with levels decreased to 36% for PFOS and 46% for PFOA exposure [18]. Therefore, exposure to PFOA and PFOS significantly inhibits capacitation in spermatozoa. Without proper capacitation, fertilization will not occur. 

To test the mechanism of capacitation inhibition, looking specifically at [Ca^2+^] alteration, Ortiz-Sánchez et al. evaluated a tracing process under a capacitation medium using exposed PFOS and PFOA sperm, with the same concentrations as in the previous study (Table 5) [18]. Fluorescence spectroscopy was used to determine [Ca^2+^] levels. Immediately after incubation, there was a significant increase in [Ca^2+^] in the PFOS-exposed group [18]. This level decreased between 1 and 2 h but underwent another significant increase at 3 h. Moreover, there was a final decrease at 4 h, which still maintained an overall increased concentration. A build-up of [Ca^2+^] occurred at 3 h capacitation by PFOA which obstructed crucial capacitation functions, suggesting that, although both compounds impact [Ca^2+^], PFOS acts on capacitation immediately, whereas PFOA’s effects occur later [18]. Clearly, [Ca^2+^] levels are targets for PFAS compounds and massively impact capacitation. 

An ionophore (A23187) was added to one treatment group to compare [Ca^2+^] results, as the ionophore increases [Ca^2+^] uptake, preparing the cell for the acrosomal reaction (Table 5) [18]. After A23187 was added, the concentration increased and stayed consistent. Moreover, the addition of any PFAS compound resulted in an increased [Ca^2+^] [18]. Spermatozoa exposed to PFOA and PFOS did not respond to the A23187 addition, implying that membrane dysfunction is inhibiting calcium uptake. When stimulated with 10 μg of P4, there was an increase in [Ca^2+^] in capacitated spermatozoa as well, and adding 10 M of A23187 stabilized [Ca^2+^] levels. However, when PFOA exposure was observed, P4 stabilized the [Ca^2+^] levels, but there was no cellular response to A23187 [18]. Similarly, in PFOS-exposed groups, decreased [Ca^2+^] was observed after P4 addition and no response to A23187 was noted. The entry of A23187 and P4 is inhibited after exposure to PFOS due to saturated [Ca^2+^]. PFOS and PFOA impacted membrane function by 49% and 47%, which inhibited cholesterol release [18]. Activation of the calcium channels required for capacitation and acrosome calcium uptake is correlated with PM hyperpolarization. These results show a similarity to the female response of PFAS exposure due to channel receptors being targeted. Ortiz-Sánchez et al. further explored membrane potential fluctuation to pinpoint the cause of increased calcium (Table 5) [18]. Hyperpolarization was produced by valinomycin and increasing KCl concentrations were added to stimulate repolarization. Post capacitation, increasing KCl allows for repolarization of PM after the membrane potential decreases as a result of valinomycin addition [18]. PFOS-treated samples did not repolarize after KCl addition and only responded minutely to valinomycin, while PFOA samples had no response to the valinomycin or KCl. Therefore, since the membrane potentials of PFOA- and PFOS-treated samples do not change, the damage from PFAS must be on the PM itself. Outside the area of fertility, PFAS may be impacting additional PM in other systems as well. More research needs to be conducted to determine the true impact and consequences of exposure. 

Another major process to further investigate is cholesterol efflux. Filipin staining shows fluorescent patterns of capacitated and non-capacitated spermatozoa [18]. This was used to determine the presence of cholesterol in PFAS-exposed samples. Ortiz-Sánchez et al. found a significant decline in the capacitated spermatozoa compared to the non-capacitated spermatozoa, suggesting that damage to the PM inhibits cholesterol efflux due to PFAS toxicity (Table 5) [18]. After exposure to PFOS and PFOA, the fluorescence was similar to the non-capacitated group. The ICC50 of PFOA, 1458 μM, did not decrease capacitation; however, the ICC50 of PFOS, 274 μM, significantly decreased the number of capacitated spermatozoa by 43%. PFOS and PFOA have median lethal concentrations (LC50) of 460 and 1894 nM. Additionally, half of LC50 for PFOA (950 μM) significantly decreased the number of capacitated spermatozoa by 28% [18]. 

Overall, Ortiz-Sánchez et al. showed that PFAS exposure impacts the capacitation reaction in numerous ways, all posing as a threat to fertilization. Immediately after exposure, sperm mortality was noted and continued to rise with increasing concentrations. Additionally, the capacitation reaction was inhibited by changing levels of [Ca^2+^] through membrane dysfunction. PM dysfunction also leads to cholesterol efflux, further impacting capacitation success. Acting through these different mechanisms, PFAS pose many threats to capacitation and ultimately fertilization.

**Figure 5:**
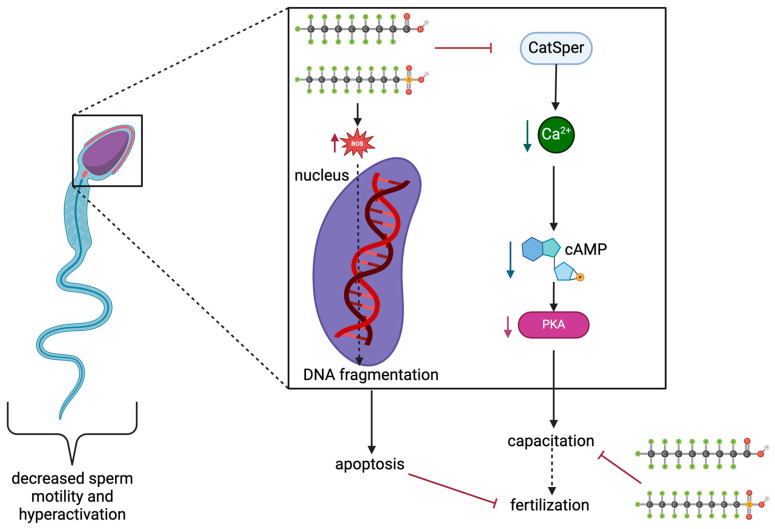
Representation of PFOS and PFOA effects on sperm capacitation. Modified from [60]. Created with Biorender.com (accessed on 2 May 2024).

Effects have also been noted in human studies. The effects of PFOA and PFOS on human sperm capacitation through cAMP/PKA mechanisms were tested by Shan et al. using concentrations up to 200 μM, the occupational average exposure (Table 5) [60]. Results are shown in Figure 5. Sperm motility and hyperactivation were significantly decreased, while sperm motility patterns notably changed as linear movements were observed rather than a typical swaying pattern. Additionally, looking at Western blot results, protein phosphotyrosine levels decreased in PFOS- and PFOA-exposed samples; and concentrations of PFOA and PFOS significantly decreased the Ca^2+^ fluorescence [60]. Decreasing Ca^2+^ levels may inhibit the cAMP/PKA signaling pathway as there was a significant decrease in cAMP levels in all concentrations of both PFOA and PFOS, except for a non-significant decrease in 10 μM of PFOA. Further proving the inhibition of cAMP/PKA signaling, there was an overall decrease in protein kinase A activation in all concentrations of PFOA and PFOS. Similar to female studies and those conducted on animals, sperm exposure to PFOS and PFOA increased ROS abundance [60]. Additionally, DNA fragmentation in the control group after capacitation was 8.43 ± 3.81%, with a significant increase to 18.76 ± 7.98%, 20.95 ± 7.14%, and 24.38 ± 7.52% detected in DNA fragmentation after 200 μM PFOS, 100 μM PFOA, and 200 μM PFOS exposure. This leads to damaged sperm nuclei and confirms that DNA damage results from high PFOA and PFOS exposure.

Clearly, consistent results of decreased capacitation are being seen in both human and animal studies. 

### 5.3. Acrosomal Reaction

**Figure 6:**
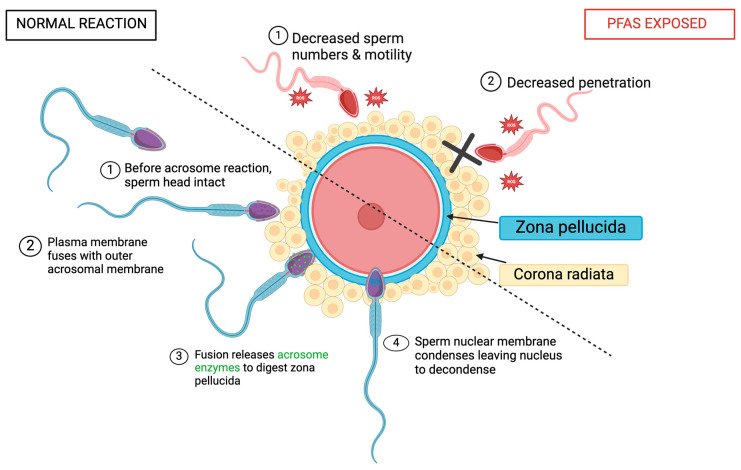
Acrosomal reaction of sperm after PFAS exposure. Created with Biorender.com (accessed on 1 May 2024).

Spermatozoa undergo an acrosomal morphological change prior to fertilization and post-capacitation to allow for conception (Figure 6) [70,71]. P4, found in the female reproductive tract, stimulates the acrosomal reaction in humans and is responsible for regulating sperm behavior [57,70]. In summary, capacitated spermatozoa bind to the ZP, acrosin penetrates through the ZP until the sperm membrane fuses with the oocyte, and fertilization occurs [42,70,71].

Looking at studies testing animal reactions, induced acrosomal reactions (iARs) were evaluated by a CTC assay conducted by Ortiz-Sánchez et al. (Table 6) [18]. They used 100, 200, and 300 µM PFOS as well as 150, 550, and 950 µM PFOA. The iAR was reduced to 1.5% by PFOS and 18% by PFOA exposure showing that PFOA has a stronger toxic effect on acrosome reactions [18]. Acrosome presence can be detected by a marker for lectin, PNA. On the other hand, PFOS exposure has a stronger effect on capacitated sperm as the 25% iAR dropped to 14%, 11%, and 1.5% (for 100, 200, and 300 μM PFOS), compared to PFOA which dropped from 27% to 24%, 23%, and 18% (for 150 μM, 550 μM, and 950 μM PFOA). Both compounds reduced the ½ CL50 levels with significant results after PFOS exposure [18].

Penetration of the cervical mucus is a key quality of accessing sperm motility. Yuan et al. analyzed 0.25, 2.5, and 25 µg/mL of PFOA and detected significant declines in the sperm that reached penetration, using the in vitro penetration test, in the 25 μg/mL PFOA exposure group (Table 6) [57]. The low-dose PFOA groups showed no change. When conducting the same experiment using 10 μM P4-induced sperm (since P4 increases sperm motility), all PFOA-exposed groups showed significant changes. The 0.25, 2.5, and 25 μg/mL groups all significantly lowered the total number of sperm that reached penetration [57]. This concludes that PFOA exposure majorly impacts penetration success. Yuan et al. used a CTC assay to analyze the acrosome reaction in spermatozoa [12,57]. Using the same dosages as above (0.25, 2.5, and 25 ug/mL PFOA), no changes were observed across all treatments. However, when P4 was used to initiate the acrosome reaction, all three exposure groups showed a significant inhibition of the acrosome reaction. Ca^2+^ is crucial for flagellar movement and CatSper, the calcium channel in sperm, is a chemosensory and polymodal channel that regulates Ca^2+^ influx [57]. CatSper is also required for penetration, fertility, and the activation of hyperactivation. P4 majorly stimulates Ca^2+^ entry through CatSper and was majorly inhibited by PFOA exposure [57]. All three concentrations of PFOA decreased [Ca^2+^] influx which ultimately decreased motility. CatSper demonstrates a clear target for PFOA damage. Briefly measuring ROS in spermatozoa, high concentrations of PFOA (25 μg/mL) significantly increased the abundance of ROS [57]. High levels of ROS can majorly affect sperm motility [12]. Yuan et al. showed that mechanisms involved in sperm motility are very susceptible to PFAS damage, which in turn impairs male fertility [38,57,58]. As well as decreasing sperm numbers, PFAS lower sperm penetration rates, directly impacting fertility chances. 

Looking at human outcomes, Shan et al. investigated AR presence using FITC-PSA staining after PFAS exposure; PFOS, PFOA, and a combined PFOS + PFOA group detected no fluorescence in the acrosome (Table 6) [60]. In the DMSO control groups, AR was measured at 53.97 ± 8.46%. For PFOS-exposed groups of 10, 100, and 300 μM, AR measurements were significantly decreased to 43.87 ± 8.97%, 38.63 ± 7.68%, and 33.60 ± 6.44 [60]. For PFOA-exposed groups of 100 and 200 μM, AR measurements were significantly decreased to 36.72 ± 2.14% and 29.09 ± 4.23%. In the combined group (100 μM PFOS + 100 μM PFOA), exposure decreased most significantly to 30.77 ± 5.32% [60]. This demonstrated that PFAS exposure can lead to decreased acrosomal reaction occurrence, further inhibiting fertilization.

## 6. Effects on Embryo Pre-Implantation

**Figure 7:**
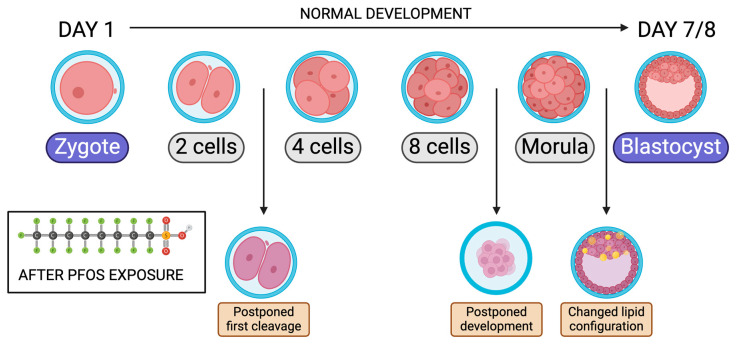
Bovine embryo development timeline from zygote to blastocyst after PFOS exposure. Modified from [78]. Created with Biorender.com (accessed on 1 May 2024).

Little information is known about PFAS’ effects in the stages between fertilization and late pre-implantation embryo development. A critical time of embryo development is the period between zygote and blastocyst (Figure 7). P4 impacts the endometrial lining by stimulating the final differentiation and secretory functions and P4 antagonists impact fertility by inhibiting ovulation and directly affecting the endometrium [72,73]. This occurs in preparation for implantation during the luteal phase. Ovarian function is disturbed as PFOA impacts P4 production. This was proven by Di Nisio et al. using UV–vis spectroscopy absorbance, while witnessing PFOA-P4 interaction in human Ishikawa cells (Table 7) [72]. Additionally, PFOA stimulation has been proven to inhibit estrogen sulfotransferase genes. Estrogen sulfotransferase inactivates estrogens and is an ideal model to assess antiprogestin effects. mRNA levels are antagonized by PFOA, impairing embryo attachment. The endometrium responds more readily to antiprogestins, such as PFOA, rather than disruption signals in the hypothalamic–ovarian axis, suggesting the strong influence that PFOA holds [72]. Decreased embryo implantation is very damaging and leads to detrimental effects on fertility. 

PCOS patients display lower embryo development while undergoing IVF treatment [23]. The etiology of PCOS is still unknown; however, many studies have found positive links to EDC exposure [74], including an association with PFAS serum levels and increased PCOS diagnosis [75]. Similarly, PFAS concentrations are significantly correlated to higher infertility rates amongst endometriosis-affected patients [76]. Looking at the maternal reproductive system, PFAS can cause reproductive disorders, further impacting the chances of getting pregnant and being able to maintain a pregnancy. 

There is a negative association between plasma concentrations of PFOA and mature oocytes as well as good-quality embryos [17,22]. A common tool to detect successful fertilization after IVF treatment is the observation of two-pronuclear (2 PN) [17]. In animal studies, Walters and Handelsman found that increased concentrations of PFOA resulted in decreased 2 PN zygotes [17]. Additionally, lipid metabolism changes lipid storage in early embryonic stages and allows for oocyte meiotic maturation [77]. Hallberg et al. looked at the 0.1 and 10 µg/mL Perfluorononanoic Acid (PFNA) effects on lipid accumulation (Table 7) [77]. After exposure to PFNA, lipid metabolism was impaired. The cumulus cloud in the 10 µg/mL group did not expand compared to the control [77]. This can be explained by the disruption of the involved PPAR receptors by lipid metabolism. Abnormal levels at this stage may produce fewer developing blastocysts. Hallberg et al. found that PFOS exposure significantly decreased the chances of the embryo reaching beyond the two-cell stage, suggesting that PFOS might have a direct effect on the embryo cleavage rate (Table 7) [78]. Using concentrations of 2 and 53 ng/mL, the 53 ng/mL group significantly decreased cleavage past the two-cell stage [78]. In bovines and humans, any delay in cleavage reflects poor embryo quality [78]. However, in later embryonic development, the delay was imperceptible. In a following study, Hallberg et al. looked at bovine COCs exposed to PFAS before fertilization (Table 7). If PFAS exposure was 10, 40, or 100 μg/mL, there was a decrease in embryos cleaving past the two-cell stage with lower concentrations of 0.01, 0.1, 1.0, and 20 µg/mL showing no change [79]. Embryos exposed to concentrations above 40 μg/mL showed a decrease in the total cell count, which reduces the chances of reaching the blastocyst stage, significantly affecting pre-implantation development [79]. 

**Figure 8:**
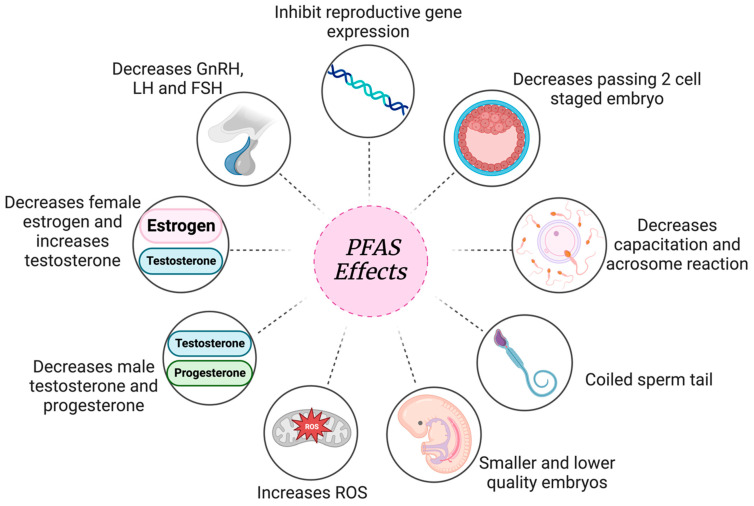
Overall look at PFAS’ reproductive effects. Created with Biorender.com (accessed on 9 January 2024).

In a third study, Hallberg et al. further examined the effects of PFOS exposure on early embryonic development (Table 7) [80]. COCs were exposed to 10 and 100 nM PFOS. PFOS-exposed zygotes had significantly higher ROS presence in the eight-cell stage, leading to impaired early embryonic development [80]. ROS presence induces apoptosis and decreases proliferation, causing a decreased blastomere count and delayed development [80]. Additionally, the proportion of inner cell mass (ICM) cells was significantly affected by PFOS exposure [80]. Proper ICM development is crucial to produce fetal structures [81]. PFOS exposure led to increased mesoderm differentiation along with decreased endoderm differentiation. This lack of balanced differentiation derives from the impaired ICM and can cause a severe embryonic lack of development. Negative outcomes on gametes after PFAS exposure, as detrimental as they are, lead to embryo attachment in order to produce a pregnancy. If exposure itself is directly impacting implantation and embryo development, this proves how dangerous PFAS exposure really could be. The result of PFAS exposure leading to hormonal inhibition, affected gametes and decreased implantation, leads to detrimental results in reproductive health (Figure 8). 

**Table 7:** Summary of findings of PFAS’ effects on embryo pre-implantation.

Species	Cell Type	In Vivo/Vitro	Endpoint	Treatment Dose	Effect Observed	Reference
Animal Studies						
*Bos taurus*	Bovine blastocysts	in vitro	Effects of PFNA on lipid accumulation in blastocysts	10 and 0.1 µg/mL PFAS	- PFNA effects lipid metabolism in blastocysts - Cumulus cloud in 10 µg/mL group did not expand - Significant difference in lipid droplets	[77]
*Bos taurus*	Bovine blastocysts	in vitro	Effects of PFOS on bovine embryonic development	2 ng/mL and 53 ng/mL of PFOS	- 53 ng/mL of PFOS significantly decreased cleavage past the 2-cell stage - Results of early delay were not noticeable in later embryonic development	[78]
*Bos taurus*	Bovine blastocysts	in vitro	Effects of PFAS on bovine COC fertilization and maturation	0.01, 0.1, 1.0, 10, 20, 40, and 100 μg/mL PFAS before fertilization and cultured for 8 days	- 10, 40, and 100 μg/mL showed a decrease in passing the 2-cell stage - 40 µg/mL + decreased total cell count	[79]
*Mus musculus*	Murine zygotes	in vitro	Effects of PFOS exposure on ICM of pre-implantation embryo	10 nM and 100 nM PFOS	- 100 nM: affects the blastocyst formation rate - Increased ROS and apoptosis - Impaired ICM in pre-implantation embryos	[80]
Human Studies						
*Homo sapiens*	Human endometrial epithelial adenocarcinoma Ishikawa cells	in vitro	Effects of PFOA exposure on progesterone in endometrial cells	0.5, 1, 1.8, 3, 5, 7, 8 mM of PFOA	- PFOA inhibits estrogen sulfotransferase genes - mRNA levels—of ITGB8, ALPPL2, and KLF5—are antagonized by PFOA, impairing embryo attachment - The endometrium responds more readily to PFOA rather than the hypothalamic–ovarian axis	[72]

## 7. Conclusions

PFAS persist in the environment and bioaccumulate in humans. Research in animal models demonstrates that they can negatively affect reproduction pathways and outcomes. There is strong evidence that PFAS affect reproductive hormones; however, the knowledge on how this occurs is limited. GnRH, LH, and FSH levels are reduced, impacting hormone synthesis, such as estrogen, testosterone, and progesterone [43]. Similarly, PFAS inhibit capacitation and acrosome reactions in sperm [18]. Although meiosis is inhibited by activating PPARγ, the strongest and most prominent impact across all species is the change in sperm morphology [31]. Coiling sperm tails are the most common and problematic effect as they prevent the sperm from travelling through the female reproductive tract and fertilizing the egg. Along with the individual effects on male and female gametes, the percentage of embryos that reach the blastocyst stage is significantly affected [79].

PFAS exposure induces negative effects on early development and reproductive functions across various species, including humans. However, most experiments were conducted using higher concentrations than the physiologically and environmentally significant ones. Altogether, the evidence in the literature strengthens the importance of reducing or ideally eliminating PFAS from the environment to prevent the accumulation in humans leading to decreased fertility and reproductive dysfunctions. Further research is needed to establish the exact levels of PFAS exposure impacting early development. Utilizing a lower consistent and chronic exposure might be more reflective of environmentally significant concentrations and more closely mimic toxicity in humans. This would properly replicate the low yet constant human exposure due to PFAS’ presence in water and diet. Infertility is increasingly becoming more common and since a link between PFAS exposure and decreased fertility is quite evident in the literature, it is crucial to further advance research and evidence leading to a stricter regulation of PFAS use, especially ultimately affecting patients undergoing in vitro fertilization procedures. 

## Figures and Tables

**Table 5:** Summary of findings of PFAS’ effects on sperm capacitation and capability competence.

Species	In Vivo/In Vitro	Cell Type	Endpoint	Treatment Dose (µM)	Effect Observed	Reference
Animal Studies						
*Sus scrofa*	in vitro	Boar spermatozoa	Effects of PFOA and PFOS exposure on sperm motility and capacitation	1000, 1500, 3000 μM for PFOS and 500, 1000, 1500, 2000, 2500 μM for PFOA	- 0 h PFOS: 20% in mortality - 4 h PFOS: 33% mortality - 1000 μM: significant decrease in capacitation - 1000 PFOS: 64% mortality - 3000 PFOS: 97% mortality - 1500 PFOA: 36% mortality - 2500 PFOA: 76.5% mortality - Capacitation levels of 64% significantly decreased to 36% for PFOS and 46% for PFOA	[18]
			Test mechanism of capacitation inhibition after exposure to PFOS and PFOA	1000, 1500, 3000 μM for PFOS and 500, 1000, 1500, 2000, 2500 μM for PFOA	- PFOS: significant increase in [Ca^2+^] at 0 h, slightly decreased between 1 and 2 h and significantly increased again at 3 h and 4 h - PFOA: increased [Ca^2+^] at 3 h	
			Compare [Ca^2+^] levels with addition of ionophore and PFOA/PFOS exposure	1000, 1500, 3000 μM for PFOS and 500, 1000, 1500, 2000, 2500 μM for PFOA	- Spermatozoa exposed to PFOA and PFOS did not respond to A23187 addition	
			Pinpoint cause of increased calcium by looking at membrane potential fluctuation	274 μM PFOS and 950 μM PFOA	- PFOS-treated samples did not repolarize after KCl and slightly responded to valinomycin - Impacted membrane function by 49% - PFOA samples had no response to valinomycin or KCl. Impacted membrane function by 47%	
			Presence of cholesterol in PFAS exposed samples	274 μM PFOS and 950 μM PFOA	- Significant decline in cholesterol in capacitated spermatozoa compared to non-capacitated - PFAS exposure caused similar results to non-capacitated spermatozoa	
			Impact of ICC50 and LC50 of PFOA/PFOS on capacitation	ICC50 of PFOA: 1458 μM ICC50 of PFOS: 274 μM LC50 of PFOA: 1894 μM (half is 950 μM) LC50 of PFOS: 460 μM	- ICC50 PFOA: did not decrease capacitation - ICC50 of PFOS: significantly decreased capacitated spermatozoa by 43% - Half of LC50 PFOA significantly decreased capacitated spermatozoa by 28%	
Human Studies						
*Homo sapiens*	in vitro	Human spermatozoa	PFAS’ effects on capacitation through cAMP/PKA mechanism and DNA damage	10, 100, and 200 μM PFOS 10, 100, and 200 μM PFOA 100 μM PFOS + 100 μM PFOA	- Significantly decreased sperm motility and hyperactivation - Sperm motility patterns changed - Decreased protein phosphotyrosine, Ca^2+^ levels, cAMP levels (except 10 μM PFOA), and PKA - Significant increases in ROS for 100 and 200 μM PFOS and PFOA. - DNA fragmentation increased significantly - Significant increase in damaged sperm nuclei in 100 μM and 200 μM PFOA and PFOS groups	[60]

**Table 6:** Summary of findings of PFAS’ effects on the acrosome reaction and penetration capability.

Species	In Vivo/In Vitro	Cell Type	Endpoint	Treatment Dose	Effect Observed	Reference
Animal Studies						
*Mus musculus*	in vitro	Human spermatozoa	PFOA exposure impacts progesterone response in sperm	0.25, 2.5, 25 μg/mL of PFOA	- Disturbed seminiferous tubules, as well as decreased sperm number, motility, testosterone, and progesterone levels	[57]
			PFOA’s effects on sperm penetration	0.25, 2.5, 25 μg/mL PFOA	- 25 µg/mL: significant decrease in sperm reaching penetration - 0.25 µg/mL: no change - All PFOA groups had a significant decrease after 10 μM P4 was added	
			PFOA’s effects on acrosome reaction	0.25, 2.5, 25 μg/mL PFOA	- No change until P4 was used to initiate acrosome reaction. All three doses showed significant inhibition of acrosome reaction - 25 µg/mL: significantly increased ROS	
*Sus scrofa*	in vitro	Boar spermatozoa	Effect of PFOA and PFOS exposure on acrosome reaction	100, 200, 300 μM PFOS 150, 550, and 950 μM PFOA	- iAR reduced by 1.5% by PFOS and 18% by PFOA - PFOS dropped from 25% to 14, 11, and 1.5% (100, 200, and 300 μM) - PFOA dropped from 27% to 24, 23, and 18% (150, 550, and 950 μM)	[18]
Human Studies						
*Homo sapiens*	in vitro	Human spermatozoa	Investigating presence of AR after PFAS exposure	10, 100, and 200 μM PFOS 10, 100, and 200 μM PFOA 100 μM PFOS + 100 μM PFOA	- AR significantly decreased in the PFOS-exposed groups from 8.46% to 8.97, 7.68, and 6.44% (10, 100, and 300 μM) - For PFOA, significant decrease to 2.14 and 4.23 for 100 and 200 μM - Combined group had biggest effect to 5.32	[60]

## Data Availability

All figures created in Biorender.com with valid publication and licensing rights.
